# Listening deeper: neural networks unravel acoustic features in preterm infant crying

**DOI:** 10.1038/s41598-025-03098-1

**Published:** 2025-07-02

**Authors:** Yuta Shinya, Taiji Ueno, Masahiko Kawai, Fusako Niwa, Seiichi Tomotaki, Masako Myowa

**Affiliations:** 1 Graduate School of Education, The University of Tokyo, Tokyo, Japan; 2 School of Arts and Sciences, Tokyo Women’s Christian University, Tokyo, Japan; 3 Department of Pediatrics, Graduate School of Medicine, Kyoto University, Kyoto, Japan; 4 Graduate School of Education, Kyoto University, Kyoto, Japan

**Keywords:** Preterm birth, Low-birth-weight infants, Crying, Acoustic analysis, Machine learning, Deep learning, Human behaviour, Paediatric research, Computational science, Acoustics

## Abstract

Early infant crying provides critical insights into neurodevelopment, with atypical acoustic features linked to conditions such as preterm birth. However, previous studies have focused on limited and specific acoustic features, hindering a more comprehensive understanding of crying. To address this, we employed a convolutional neural network to assess whether whole Mel-spectrograms of infant crying capture gestational age (GA) variations (79 preterm infants; 52 term neonates). Our convolutional neural network models showed high accuracy in classifying gestational groups (92.4%) and in estimating the relative and continuous differences in GA (*r* = 0.73; *p* < 0.0001), outperforming previous studies. Grad-CAM and spectrogram manipulations further revealed that GA variations in infant crying were prominently reflected in temporal structures, particularly at the onset and offset regions of vocalizations. These findings suggest that decoding spectrotemporal features in infant crying through deep learning may offer valuable insights into atypical neurodevelopment in preterm infants, with potential to enhance early detection and intervention strategies in clinical practice.

## Introduction

Acoustic features of infant cries have been studied as a noninvasive tool to assess neurophysiological stress and developmental traits in the medical field as well as an emergent signal for conveying them to caregivers^1^. Abnormal acoustic features of infant crying have been linked to various pathological conditions^2^, including neurological disorders^3^ and developmental delays^4–6^. This association suggests that acoustic features of early infant crying may serve as indicators for autonomic dysfunction and predictors of developmental delays in the social and language domains in high-risk infants^5–8^. Combining cry acoustics with other behavioral indicators (e.g., limb movements, eye movements) may further enhance developmental predictions^9,10^. Therefore, a deeper understanding of infant cry acoustics in relation to neurodevelopment is crucial for advancing clinical applications in high-risk infants.

Preterm infants represent a particularly relevant high-risk group for gaining further insights into this issue. According to several cohort studies and meta-analyses, preterm birth is associated with increased risks of developmental delays related to cognitive, language, and social functions^11–16^. Research also suggests that preterm infants exhibit atypical cry patterns, such as higher *F*_0_ in pain-induced^17,18^ and spontaneous cries^19^ around term-equivalent age compared with term neonates. Higher *F*_0_ has also been shown to be partially associated with lower gestational age (GA)^20^ and respiratory sinus arrhythmia (i.e., high frequency of heart rate variability), suggesting that the *F*_0_ features of voices reflect parasympathetic dysfunction in preterm infants^7^. Furthermore, smaller *F*_0_ variation in infant cries has been reported to predict worse language and cognitive outcomes, particularly expressive language skills^5,6^, suggesting that the acoustic analysis of infant cries has the potential to assess neurodevelopment. However, the future application of cry acoustics in clinical practice requires addressing several challenges related to the identification of more valid and reliable acoustic features specific to preterm infants in order to assess their neurodevelopment.

One of the major challenges in cry acoustics research is the limited scope of previous studies that have predominantly focused on a specific set of acoustic features, particularly *F*_0_ abnormalities (e.g., high/low *F*_0_)^2,20,21^. This narrow focus may hinder a more comprehensive understanding of the relationship between cry acoustics and neurodevelopment in high-risk infants such as preterm infants. Anatomical constraints of the vocal tract in infants younger than 3 months of age make researchers emphasize *F*_0_ features^22,23^, individual variations in crying may be more discernible in *F*_0_, which operates independently of the vocal tract and is determined by the oscillation of the vocal folds. Nevertheless, a variety of other acoustic parameters such as melodic contour^5,6,24^, formant frequency^23,25^, biphonation^26,27^, and noise concentration^28^ also hold diagnostic potential, albeit individually rather than comprehensively. Therefore, leveraging machine learning to incorporate a broader range of cry acoustic features and extract the corresponding higher-order statistical patterns may provide a more comprehensive and unbiased approach for identifying high-risk pediatric populations, such as preterm infants.

The application of machine learning methods to infant cries surpasses traditional approaches in classifying neonatal vocalizations^29^, detecting respiratory pathologies^30^, identifying cry reasons^31^, and cry detection^32^. In particular, recent advances in deep neural networks (i.e., deep learning), such as convolutional neural networks (CNNs), have enabled the analysis of whole Mel-spectrograms for cry recognition. This shift from predefined acoustic parameters to full-spectrum analysis allows for the extraction of richer and more complex acoustic patterns relevant to neurodevelopment. Orlandi et al. demonstrated that employing 10 acoustic parameters, the Random Forest method can classify preterm and term infants with approximately 87% accuracy^33^. However, this approach relies on a limited set of acoustic features, such as *F*_0_ variables, which may constrain a more comprehensive understanding of preterm infant crying patterns. To date, no study has applied CNN-based full spectrogram analysis to distinguish preterm infants cries from those of term infants. In addition, further advances in inspecting the computational mechanisms of CNN models, such as gradient-weighted class activation mapping (Grad-CAM^34^), may help identify novel acoustic features particularly relevant to high-risk populations such as preterm infants.

In the context of preterm infants, GA at birth is a key predictor of neurodevelopmental outcomes, with lower GA being associated with increased risks of cognitive, language, and motor delays^11–16^. GA also influences various behavioral and physiological markers, including cry acoustics [e.g., melodic features] and autonomic function^5,7,19^. Given these findings, understanding how fetal maturation shapes cry acoustics at birth requires isolating the effects of GA from postmenstrual age (PMA), which reflects the cumulative effects of prenatal and postnatal experiences at the time of cry recording. By controlling for PMA, researchers can determine whether cry acoustics reflect developmental differences associated with fetal maturation rather than postnatal experience.

This study explores the potential of CNN-based approaches for distinguishing cry spectrograms of preterm and term infants at term-equivalent ages (i.e., PMAs ranging from 35 to < 42 weeks), specifically focusing on (1) improving classification accuracy and (2) identifying relative and continuous differences in gestational weeks (i.e., not binary) based on cry features. By addressing these objectives, we aimed to overcome the limitations of previous studies on preterm infants (e.g.^18,19,33^) that relied on manually selected acoustic parameters, such as *F*_*0*_ features. This study did not directly examine the relationship between cry acoustics and neurodevelopment or clinical applications in preterm infants; however, its findings may contribute to these areas by providing a more objective and comprehensive approach for analyzing infant cries.

Beyond classification accuracy, it is essential to understand the acoustic features driving model performance. If deep learning model can differentiate between earlier infants’ GA from later ones via Mel-spectrograms of cries, this implies that meaningful acoustic features underlie these differences. Thus, an additional aim was to clarify the acoustic features underlying the high classification performance. Of particular interest were temporal fluctuations in acoustic features (e.g., melodies); these features correlate with developmental outcomes, specifically in the language domain^7,8^. Therefore, we further examined this possibility by experimentally “losing” such features from the Mel-spectrograms during the network training. The x-axis (i.e., temporal information) of the spectrograms was shuffled (shuffle condition), and the shuffled spectrograms were further sliced into shorter time windows (shuffle and slice conditions). These comparisons allowed us to assess the extent to which time-series information on frequency changes contributed to this group classification. Furthermore, this study seeks to enhance the interpretability of deep learning models by utilizing advanced interpretable methods such as Grad-CAM^34^. This approach enables the visualization of the specific spectral regions that are most influential in classification, offering a more transparent and interpretable framework for identifying the acoustic features that distinguish preterm and term infants.

Therefore, using CNN-based spectrogram analysis, this study provides a more objective and comprehensive characterization of infant cries in relation to developmental risk factors (e.g., preterm birth, GA), which may lead to a better understanding of neurodevelopmental processes in this high-risk population. These insights may ultimately support early detection and improve developmental risk assessment strategies for preterm infants in clinical settings.

## Results

### Understanding the differences in gestational group captured in Mel-spectrograms of infant crying

#### Model and evaluation approach

**Fig. 1:**
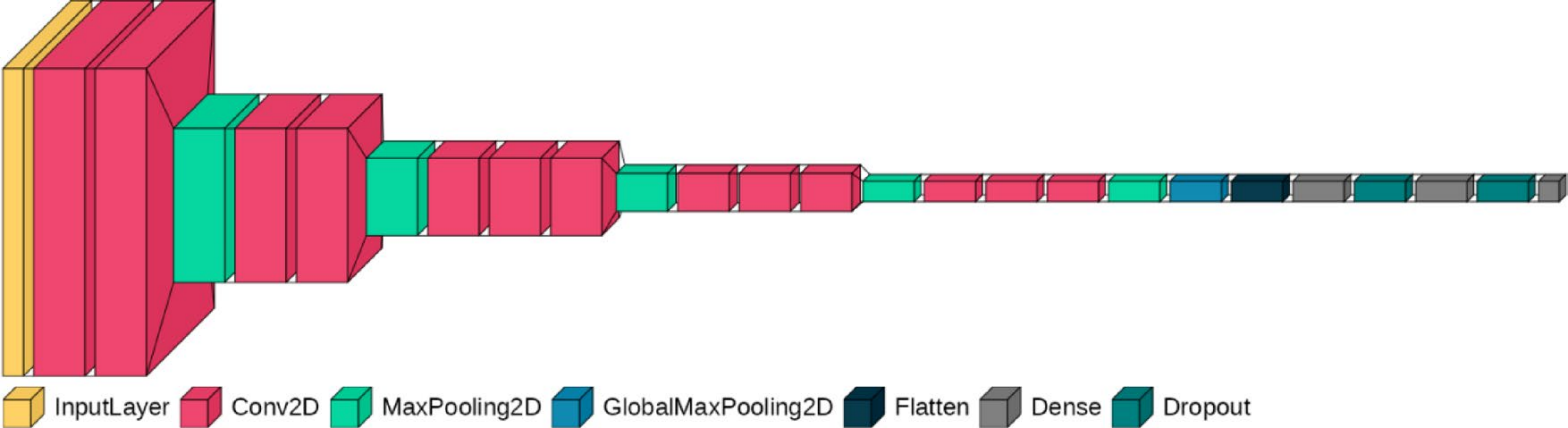
Modified VGG16 convolutional neural network (CNN) architecture for transfer learning. Processes 200 × 300 pixel images, with ImageNet pre-trained weights, frozen convolution blocks, two added hidden layers (relu and 25% dropout) and a final two-unit classification layer.

Figure 1 shows the architecture of the CNN classification model. We utilized the base layers of the VGG16 architecture^35^ for feature extraction. These layers were succeeded by two consecutive dense layers, culminating in an output layer consisting of two units for classification. The target vector was set as [1, 0] to signify the cry Mel-spectrogram of preterm infants and [0, 1] for term infants.

The dataset comprised 131 infants (preterm, *n* = 79; term, *n* = 52) with 184 cry recordings (preterm, *n* = 132; term, *n* = 52). The number of recordings per infant varied from one to five (*M* = 1.67, *SD* = 0.92) in the preterm group, whereas term infants were recorded only once. Considering that multiple cry samples were obtained from a single infant, our approach can be described as multi-instance learning. However, this deviates from the conventional multi-instance learning scenario in that every instance (or cry) in our study was assigned a specific target label. The ideal result in this scenario is the capability of the model to classify each individual cry input. Thus, a single trial in our model refers to the presentation of a single Mel-spectrogram to determine its gestational category, making the number of trials per epoch equal to the total number of available cries.

To mitigate potential biases, we applied leave-one-infant-out cross-validation, ensuring that no recordings from the same infant appeared in training and testing sets. Specifically, in each iteration, all cry instances from a single infant were excluded from the training dataset and were reserved exclusively for model evaluation. This approach prevented overfitting and ensured model generalization. This procedure was repeated for all 131 infants in the dataset. For an in-depth understanding of the dataset and training process, see the “Methods” section.

#### Classification performance of preterm versus term groups on CNN

**Fig. 2:**
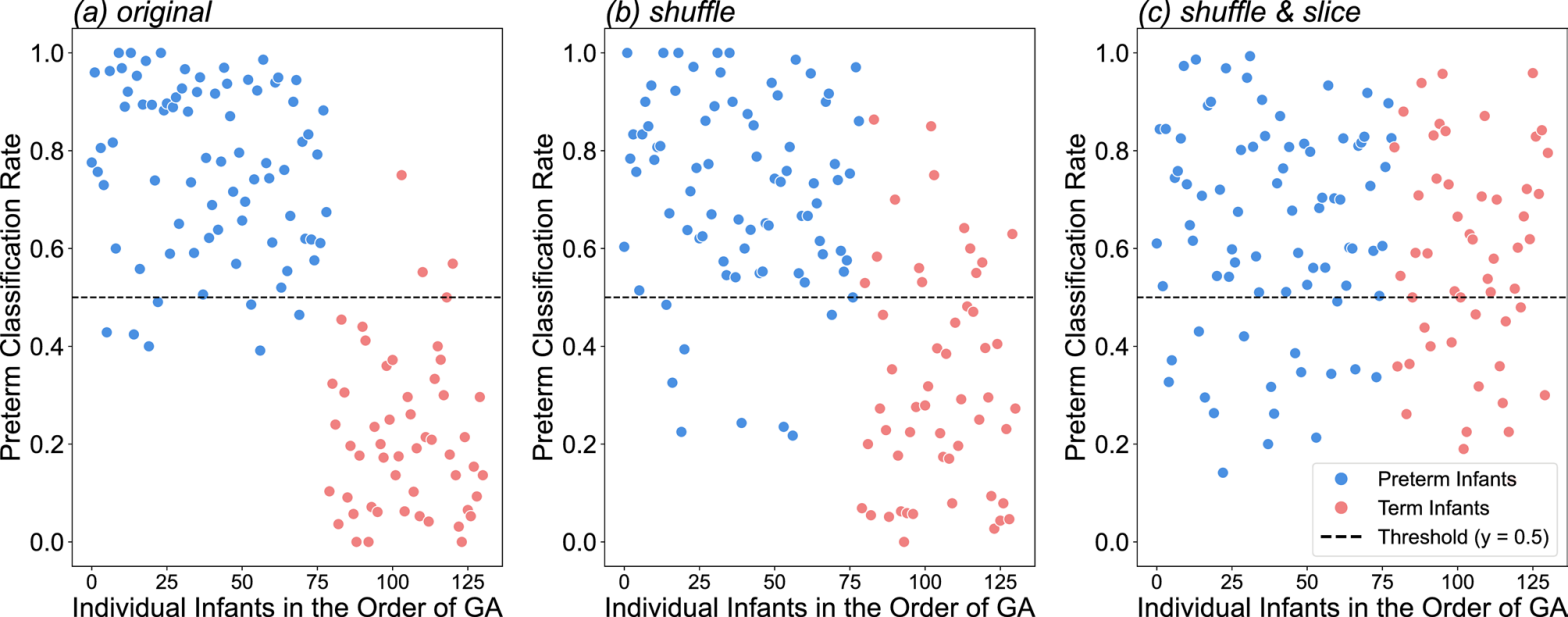
Classification plots for preterm status under three distinct conditions (original, shuffle, shuffle and slice). Each point represents an infant ordered by gestational age (GA). Blue points indicate preterm (n = 79), and red points denote term infants (n = 52). The dashed line marks a 0.5 classification threshold.

Figure 2a presents the classification plots that distinguish between preterm and term infants. Each plot represents an individual infant, arranged according to GA, with colors indicating true classes. The y-axis represents the ‘preterm classification rates’. This rate was calculated by dividing the number of cry instances identified as ‘preterm’ by the total number of cry instances of the infant. Consequently, if this rate exceeds/falls below 0.5 (as represented by the dotted vertical line indicating the chance level), the infant is classified as preterm/term, respectively. Of 131 infants, 120 were correctly categorized, resulting in an accuracy rate of 92.40%. This performance surpasses that of the model presented by Orlandi et al.^33^, although we incorporated all cry instances from each infant into our analysis.

#### Significance of temporal features in cry analysis

Mel-spectrogram capture temporal fluctuations (such as contour and variance) in acoustic features, allowing the network to improve its classification skills. To assess the significance of temporal features, we repeated the entire modeling and evaluation process under two distinct conditions: (1) using shuffled Mel-spectrograms and (2) using shuffled and sliced Mel-spectrograms. In the first scenario, the x-axis values (representing time) within each Mel-spectrogram were randomized, leading to the loss of frequency contour information over time. In the latter scenario, each shuffled Mel-spectrogram was segmented into one-tenth sections along the horizontal axis. This not only disrupted the frequency contour but also the distributional data (e.g., variances) within each input. If temporal features play a pivotal role, the classification outcomes under these conditions should decline.

**Fig. 3:**
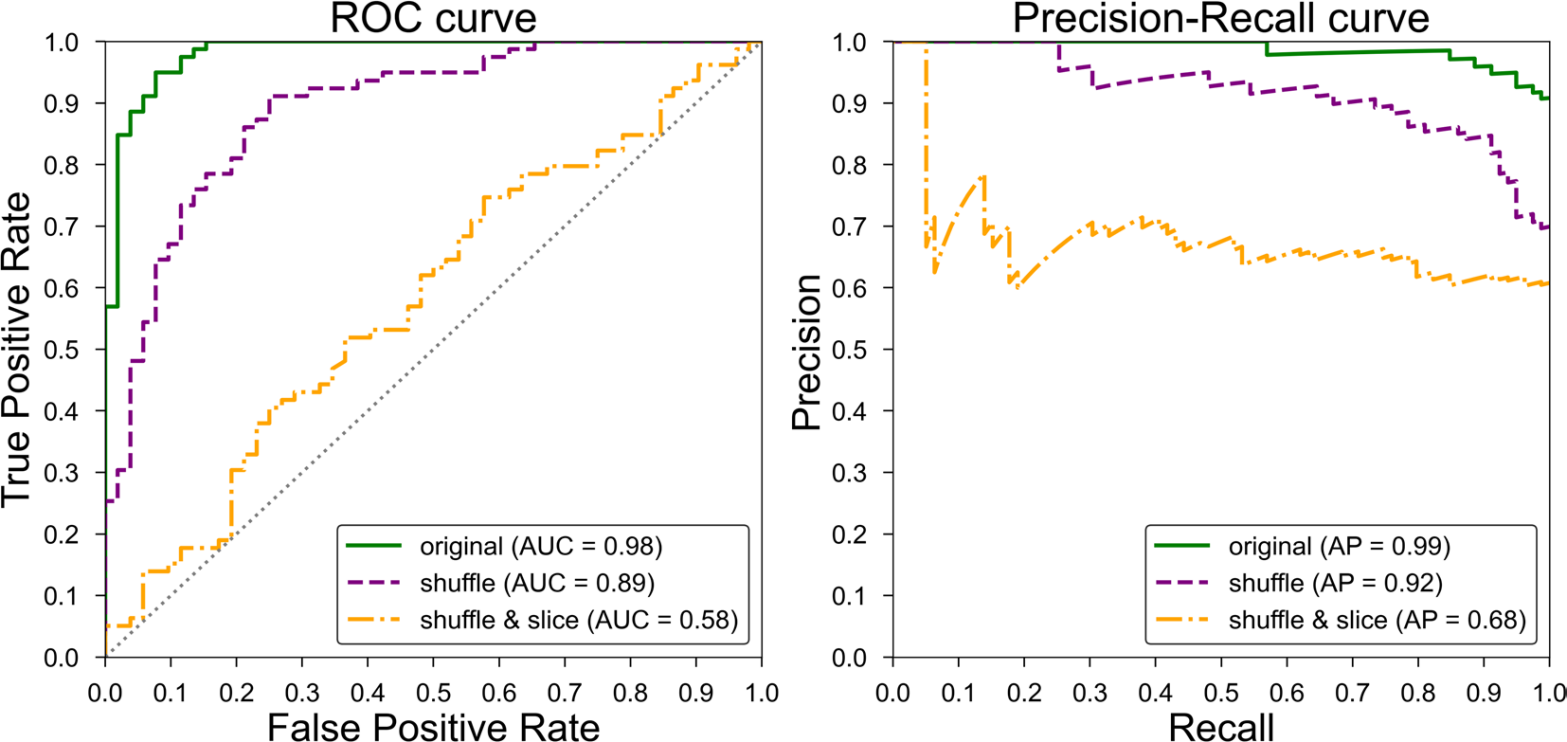
ROC and precision–recall curves of the CNN model’s classification for gestational group under three distinct conditions (original, shuffle, shuffle and slice). The areas under the curve (AUC) and Average precision (AP) provide a measure of model performance for each condition.

As shown in Fig. 2b and c, fewer infants were correctly categorized in the shuffle, and shuffle and slice conditions. Table 1 lists the accuracy, precision, recall, and F1 metrics for each scenario. One-sided binomial tests assessing the accuracy of the model for each condition revealed that all models significantly outperformed chance (original: *p* < 0.0001; *h* = 1.011; shuffle: *p* < 0.0001; *h* = 0.747; slice: *p* = 0.0004; *h* = 0.239). Figure 3 shows the receiver operating characteristic (ROC) and Precision-Recall curves for each condition. The area under the ROC curve (AUC) for the original condition was 0.98, which was higher than the AUCs for the shuffle (0.89) and slice (0.58) conditions.

**Table 1:** The accuracy, precision, recall, F1 score of CNN model in identifying preterm infants’ cries from term ones.

Condition	Accuracy (%)	Precision (%)	Recall (%)	F1 score (%)
Original	92.4	96.0	91.1	93.5
Shuffle	83.2	84.3	88.6	86.4
Shuffle and slice	61.8	65.3	78.5	71.3

Although the ROC curve distinctly highlighted the disparities between the three conditions, we deemed it essential to conduct a comprehensive statistical analysis. Specifically, we conducted a repeated-measure analysis of variance (ANOVA) with “condition” (original vs. shuffle vs. shuffle and slice) as a within-participant factor for the model’s accurate classification rate of each participant’s cries (i.e., continuous variable). The ANOVA showed a significant main effect of condition (*F*_2, 260_ = 49.604, *p* < 0.0001). Post hoc testing using the Bonferroni method revealed that the classification rate of the original condition (*M* = 0.77, *SD* = 0.17) was significantly higher than that of the shuffle condition (*M* = 0.70, *SD* = 0.21; *t* = 5.155, *p* < 0.0001) or slice condition (*M* = 0.55, *SD* = 0.24; *t* = 8.943, *p* < 0.0001). In addition, we found a significant difference between the shuffle, and shuffle and slice conditions (*t* = 5.498, *p* < 0.0001). These patterns underscore the significance of the frequency contours and variance inherent in the Mel-spectrogram for effective classification.

#### Grad-CAM: highlighting key features within Mel-spectrograms

The juxtaposition of the original condition with the shuffle, and shuffle and slice conditions underscores the pivotal role of temporal features in a single Mel-spectrogram of infant crying. To delve deeper into this aspect, we applied the grad-CAM technique^34^ on the terminal convolution layer of the original network. This method provides a visualization that accentuates the salient regions of the Mel-spectrogram, shedding light on the areas that the model prioritizes during its sophisticated classification.

**Fig. 4:**
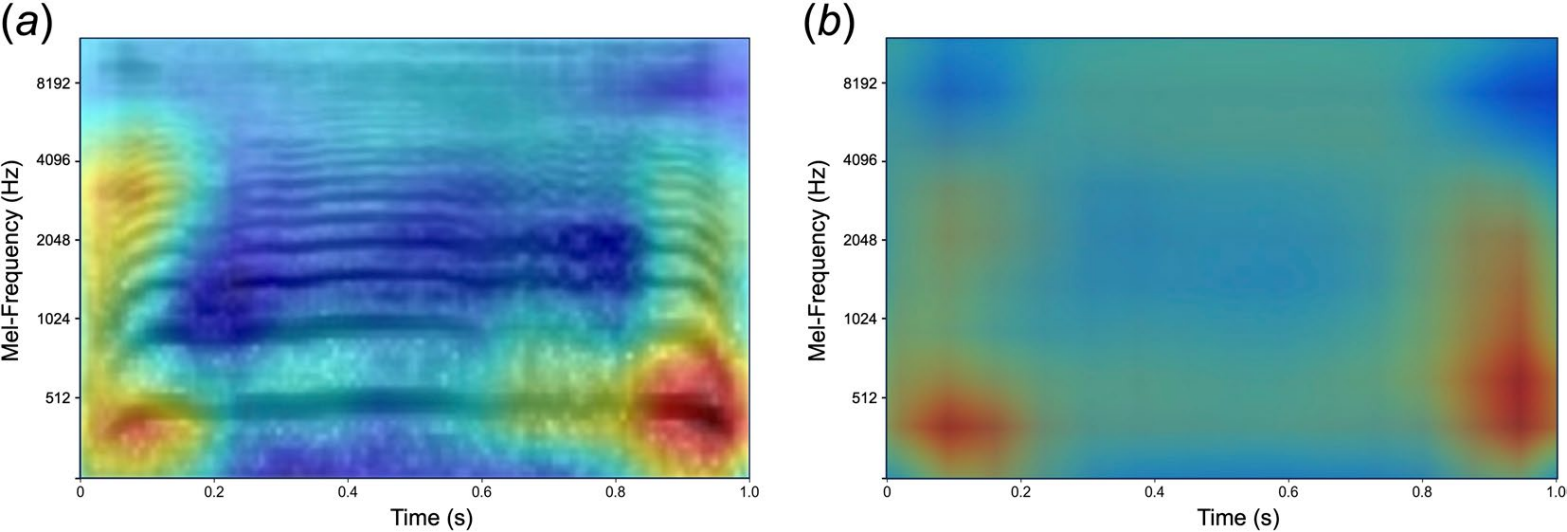
(**a**) An example of Grad-CAM visualization applied to a correctly classified Mel-spectrogram of infant crying. (**b**) The average across all correct instances, standardized to a resolution of 200 × 300 pixels. Brighter areas denote regions of increased importance, suggesting that these spectral-temporal patterns were instrumental for the CNN model’s classification.

The left panel of Fig. 4 shows an example of Grad-CAM visualization applied to a correctly classified Mel-spectrogram of infant crying. The illuminated regions highlight the spectral-temporal patterns that the CNN model took into account during the classification process. To discern common areas across all correctly classified Mel-spectrograms, individual Grad-CAM output heatmap values were averaged and displayed in the right panel. This revealed that the pivotal regions for classification were temporally clustered in the early (0–20%) and late (80–100%) phases of vocalization. Frequency-wise, these essential areas predominantly reside in the low-frequency domain (e.g., < 1024 Hz) and are aligned with the fundamental frequency. However, the model leverages high-frequency regions for its predictions. These findings imply that although specific temporal segments of the infant cry spectrogram are vital for preterm birth classification, a diverse array of regions also play a role. This aligns with the conclusions drawn earlier, reinforcing the importance of temporal features of infant crying in the classification process.

### Understanding the relative and continuous GA differences captured in Mel-spectrograms of infant crying

#### LSTM network model

Thus far, our results demonstrate that Mel-spectrograms of infant crying provide sufficient information for binary GA classification. To further validate our approach, we explored estimations that transcend binary categorizations. Our focus shifted to discerning whether the Mel-spectrograms captured the relative and continuous age variations. If this is the case, a deep learning model should be adept at gauging relative GA differences, essentially undertaking a regression task to estimate a continuous measure. This approach yielded a more robust and insightful model. Our objective was to gauge relative and continuous GA variations, rather than to predict the exact GA. Therefore, we anticipated a positive correlation between the model’s estimated GA for each infant and the actual observed GA.

**Fig. 5:**
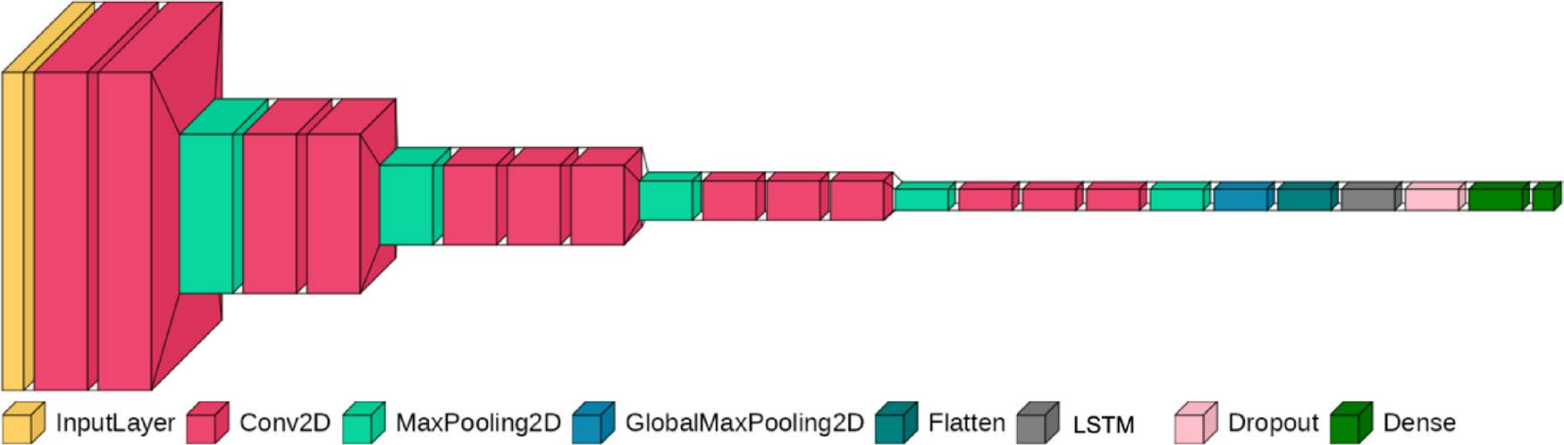
CNN model with a long short-term memory (LSTM) layer designed to estimate gestational age (GA) from sequential cry instances.

To circumvent the issue of many-to-one mapping, where one of the various cry patterns from a single infant can be selected to estimate a consistent GA in each trial, we adopted the LSTM (long-short-term model) network^36^, as depicted in Fig. 5. This model is designed to assimilate sequential inputs from all Mel-spectrograms of an individual infant to produce a singular output representing the GA. We possessed timestamps for each cry instance, facilitating their arrangement in a chronological sequence for a structured presentation.

For evaluation, we followed the leave-one-out method. In this approach, Mel-spectrograms from 130 infants formed the training set, whereas data from one excluded infant served as the evaluation set. This procedure was systematically repeated 131 times to ensure that each infant was selected as a test subject at least once.

**Fig. 6:**
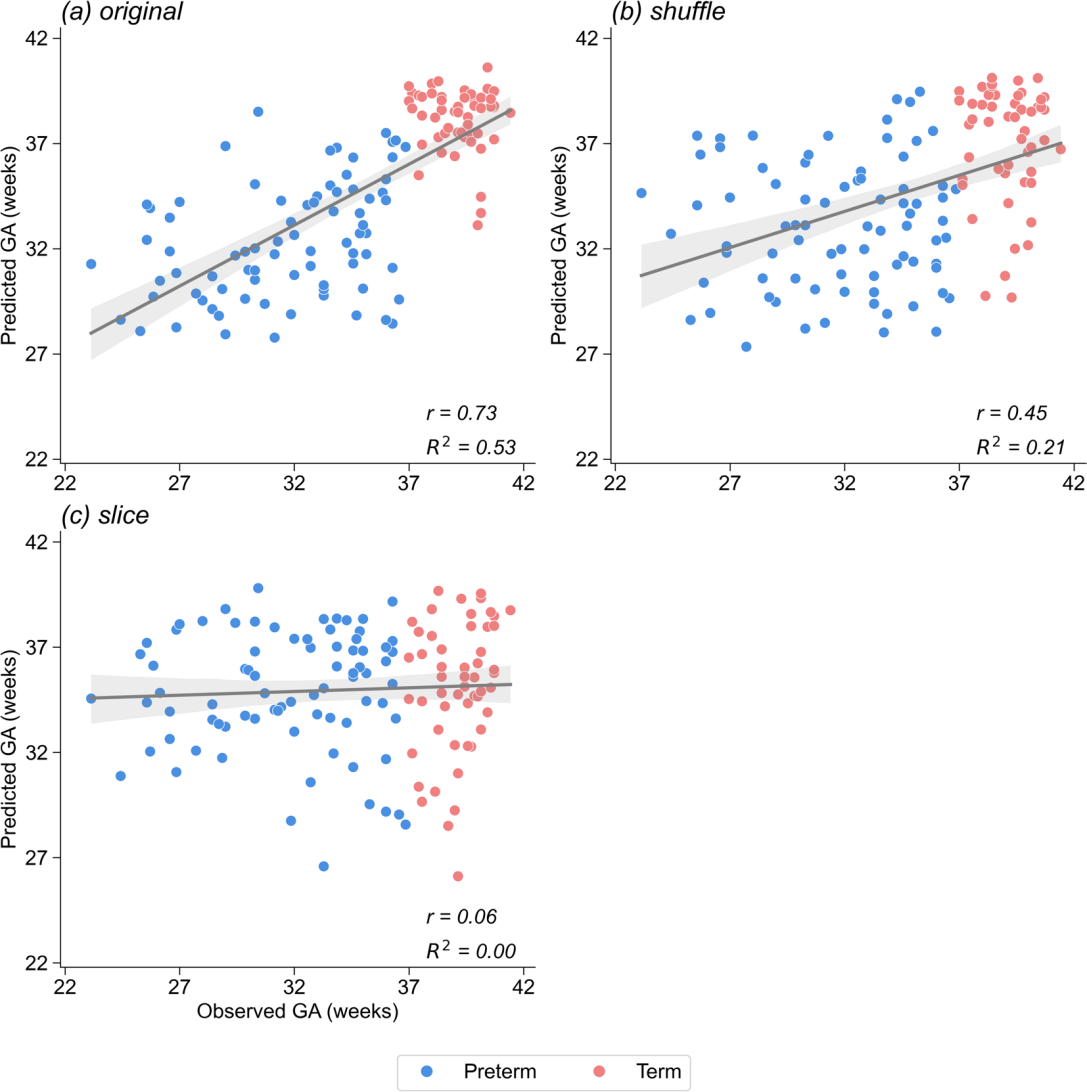
Scatter plots and regression lines with 95% confidence intervals for the relationships between the observed and estimated gestational age (GA) by the LSTM network model under three distinct conditions (original, shuffle, shuffle and slice) for all participants (n = 131).

Figure 6a shows a scatterplot of the observed GA and the GA estimated by the LSTM network derived from the sequential cry spectrograms of each infant. Correlation analysis revealed a strong positive association between the observed and estimated GA in the original condition (*r* = 0.73, *R*^2^ = 0.53, *p* < 0.001; Fig. 6a). This underscores the capability of the Mel-spectrograms of infant crying in detecting nuanced variations in GA.

Conversely, we conducted the modeling and evaluation process using the shuffled Mel-spectrograms (as illustrated in Fig. 6b) and the shuffled-and-sliced Mel-spectrograms (depicted in Fig. 6c), mirroring the approach taken with the CNN model earlier. Under these altered conditions, there was a moderate yet significant correlation between the observed and estimated GA in the shuffle scenario (*r* = 0.45, *R*^2^ = 0.21, *p* < 0.001; Fig. 6b). However, this correlation was not significant in the shuffle and slice scenarios (*r* = 0.06, *R*^2^ = 0.00, *p* = 0.531; Fig. 6c).

For a comprehensive statistical analysis, we evaluated differences in correlation coefficients using the methodologies proposed by Pearson and Filon^37^ and Zou^38^. The correlation coefficient of the original condition (Fig. 6a) was significantly different from that of the shuffle condition (Pearson–Filon *z* = 4.665, *p* < 0.001; Zou’s CI 0.166–0.403). A similar significant disparity was observed between the original, and shuffle and slice conditions (Pearson–Filon *z* = 7.300, *p* < 0.001; Zou’s CI 0.491–0.857). Moreover, a direct comparison between the shuffle and, shuffle and slice conditions also revealed a significant difference (Pearson–Filon *z* = 3.884, *p* = 0.0001; Zou’s CI 0.192–0.597). These findings further underscore the indispensable role of temporal fluctuations in infant crying when estimating GA.

Under the original condition (Fig. 6a), one may argue that the positive correlation reflects a binary group difference in GA (i.e., preterm and term groups) rather than a graded linear relationship with GA. However, our subgroup analysis indicated that the correlation remained significant in the original condition when only preterm infants (i.e., blue plots in Fig. 6a) were subjected to the analysis (*r* = 0.366, *R*^2^ = 0.13, *p* < 0.001), suggesting that their crying does indeed capture continuous differences in GA. The correlation was not significant when only the term group was subjected to the analysis under this condition (*r* = − 0.129, *R*^2^ = 0.02, *p* = 0.362); however, this could be due to the ceiling effect. Furthermore, the correlations were also non-significant in the shuffle and shuffle and slice conditions (Fig. 6b,c), regardless of which group of infants were subjected to the split analysis. Therefore, the original cry Mel-spectrogram may capture continuous differences in GA, at least within the range of the preterm group (i.e., GA < 37 weeks).

Although there was a positive correlation, the observed and estimated GA values were not exactly the same (i.e., there were many plots on the upper side of the diagonal y = x line), but this is easily explainable. First, the main aim of the current simulation was to gauge the relative and continuous GA variations and not to predict the exact GA, which would be unrealistic to expect solely from infants’ crying. Second, the distribution of the GA values in the current dataset were left-skewed (i.e., skewness = − 0.54, *M* = 34.66 weeks, *SD* = 4.63, range = [23.14, 42.42]), under which the output value of the network was naturally biased towards a higher value. This tendency is particularly clear when evaluating a generalized dataset that was not included in the training set. Therefore, the estimated GA were not exactly the same as the observed GA.

## Discussion

This study explored the potential of CNN-based spectrogram analysis for distinguishing between preterm and term infant cries, with a focus on (1) improving classification accuracy and (2) identifying relative and continuous differences in GA based on cry features. By leveraging deep learning, we sought to extend previous research that primarily relied on manually selected acoustic features, such as *F*_*0*_. Additionally, we examined the role of temporal fluctuations in cry acoustics and identified key spectrotemporal regions that contribute to the classification, providing new insights into the acoustic basis of early neurodevelopmental differences and clinical applications in preterm infants.

### CNN-based classification and GA estimation

The results demonstrated that the CNN model may provide a relatively higher accuracy (92.4%) in distinguishing preterm and term infant cries compared with previous machine learning-based models, such as Orlandi et al. (87%)^33^. Importantly, this classification performance was obtained under the more challenging conditions than the past study: specifically, (1) we implemented a rigorous generalization test, excluding all cry instances of a single infant from the training set, and used them for the generalization test; (2) our study included a larger number of infants (131 vs. 38) and crying stimuli (6523 vs. 3324); and (3) we analyzed all cry instances rather than a subset, whereas Orlandi et al.^33^ randomly excluded more than two-thirds of the cry instances, though the impact of this exclusion was not reported. The enhanced classification performance of our model may be attributed to our use of entire Mel-spectrograms instead of specific acoustic feature vectors and our selection of CNN models capable of analyzing entire Mel-spectrograms.

Beyond binary classification, we explored whether our model, which combined an LSTM layer with a CNN structure, could estimate relative and continuous differences in gestational weeks from infant crying. A significant correlation was observed between actual GA and the estimated GA of the model (*r* = 0.73,* R*^2^ = 0.53). Furthermore, subgroup analysis indicated that this correlation remained significant in preterm infants (*r* = 0.366, *R*^2^ = 0.13), even after excluding term infants from the analysis to account for the possibility that the effect was driven solely by binary difference. This result extends previous findings by Shinya et al.^19^, who reported a weaker association between GA and the fundamental frequency (*F*_*0*_) of cries (*n* = 64; *r* = − 0.48 for maximum *F*_*0*_). Given that GA is one of the strongest predictors of developmental outcomes for preterm^11^ and even for term infants^39^, the ability of our CNN-based spectrogram analysis to identify acoustic features predictive of GA represents an important step forward. These findings may suggest the potential of deep learning models for capturing GA-related acoustic characteristics. Further investigation is needed to determine whether cry-based models can reliably capture relevant acoustic markers of early neurodevelopment.

### The role of temporal structures in GA classification

Through experimental manipulation, we demonstrated that temporal structures (e.g., melody and rhythm) of cry acoustics play a crucial role in distinguishing between preterm and term infants. Our results revealed that when the spectrograms of cries were randomly shuffled along the time axis to disrupt their temporal variation (i.e., shuffle condition), the classification accuracy of the CNN model declined by 9.2% compared with the original. Furthermore, the accuracy further decreased by 13% from the original model when the shuffled spectrogram was further sliced along the time axis, causing a loss of the temporal distribution features within a single spectrogram (i.e., shuffle and slice conditions).

Similar reductions in performance were also evident when the relative and continuous differences in GA were estimated. These findings align with prior work showing that melodic complexity and variability in crying are associated with GA and later language development^5,6^, supporting the idea that the temporal structures of cry acoustics may be essential for capturing neurodevelopmental differences. 

### Grad-CAM analysis: key spectro-temporal regions for classification

Grad-CAM analysis provided additional insights into the spectrotemporal regions that contributed to classification performance. The most salient regions were concentrated in the onset (0–20%) and offset (80–100%) of cries, with low-frequency regions (< 1024 Hz), including *F*_*0*_, playing a major role in classification. Additional contributions were observed in the mid-to high-frequency bands, suggesting that non-*F*_*0*_ components may also encode gestational information. These findings align with research on prosodic structures in spoken language, where utterance onsets and offsets have been shown to be the most acoustically and phonologically informative regions owing to their prosodic prominence^40^. In spoken language, these regions are crucial for syntactic and discourse structuring as well as for signaling prosodic boundaries and prominence. The parallels observed in infant cries suggest that similar temporal structuring may emerge before linguistic experience, potentially serving as an early developmental precursor to speech organization.

Furthermore, the prominence of these spectrotemporal regions in classification suggests that gestational differences between preterm and term infants are not solely captured by fundamental frequency variations but also by higher-order temporal patterns embedded in the cry structure. The manner in which the frequency components fluctuate over time, particularly within the onset and offset phases of vocalization, may reflect the underlying neurophysiological differences in auditory-motor integration. Thus, these results also suggest the importance of analyzing whole spectrograms rather than focusing on isolated acoustic parameters, as they may allow for the detection of complex, temporally structured cues that could be crucial for understanding early neurodevelopmental trajectories regarding spoken language development.

### Implications for neurodevelopment and clinical applications

The identification of key spectrotemporal regions in infant cries suggest that early vocalizations may reflect the underlying neurophysiological maturation in preterm infants. Given that early crying is one of the first communicative behaviors, its acoustic properties may be shaped by the developing nervous system and may provide a window into individual differences in neurodevelopmental trajectories. The observed role of temporal fluctuations (i.e., contour and variance) of frequencies in the estimation of GA is consistent with prior studies, indicating that individual differences in cry melodies could be linked to later language acquisition^5,6^. In addition, the observed prominence of the onset and offset regions in the classification may support the idea that the temporal structuring of cries share fundamental organizational principles with prosodic features in speech and language processing^40^.  Therefore, further unraveling the spectrotemporal features of infant crying using deep learning may contribute to a deeper understanding of how the auditory-motor experience in early vocalizations contributes to later speech and language development.

From a clinical perspective, these findings highlight the potential of cry analysis as a noninvasive tool for identifying preterm infants at risk of developmental delays. Unlike traditional assessments that require specialized expertise, such as neurological examinations or behavioral coding, automated analysis of cry spectrograms could potentially serve as an accessible screening tool in neonatal care settings, such as in cases of respiratory injury^30^. By incorporating an analysis of prosodic-like temporal patterns, future cry-based screening tools may improve the sensitivity and specificity of risk detection, thereby contributing to earlier intervention strategies. However, significant challenges remain in translating these findings into clinical applications. For instance, the extent to which cry-based models can improve risk detection beyond existing assessment methods is uncertain, and further validation is required. Longitudinal studies integrating cry analysis with other developmental markers, such as spontaneous movements, facial expressions, and respiratory patterns, may provide a more comprehensive understanding of early neurodevelopment and improve risk assessment approaches in clinical practice^9,10^.

### Limitation

Although this study provides promising insights into the potential of deep learning-based Mel-spectrogram analysis for distinguishing preterm and term infant cries and estimating GA, some limitations must be considered.

First, the dataset used in this study was limited to a single set of recordings from a specific population. In particular, preterm infants have a wide range of diverse backgrounds and medical conditions that may influence their cry acoustics. Therefore, generalizing these findings to a broader population of preterm infants remains challenging. Future studies should examine more diverse datasets, including infants from different linguistic, cultural, and clinical backgrounds, to better assess the robustness of these models in various populations.

Second, although deep-learning methods, particularly CNN-based models, are powerful tools for capturing complex patterns in acoustic data, the interpretability of such models remains challenging. Experimental conditions such as shuffled, and shuffled-and-sliced Mel-spectrograms were implemented to assess the impact of the temporal structure. However, these manipulations may introduce variability, which requires more rigorous validation. Additionally, although Grad-CAM provided some insights into the spectrotemporal regions most relevant for classification, further validation is required to confirm whether these regions correspond to biologically meaningful features. More interpretable models or complementary analytical approaches, such as explainable AI (XAI) methods (for example)^41,42^, could improve our understanding of the acoustic components driving model predictions.

Finally, although we observed significant associations between cry acoustics and GA, we did not establish causality or mechanistic explanations for these relationships. The observed acoustic differences may be influenced by multiple developmental and environmental factors beyond GA. Future longitudinal studies incorporating neurophysiological and behavioral assessments could help disentangle the contributions of GA, postnatal experience, and broader neurodevelopmental processes.

## Conclusion

This study demonstrated that a full Mel-spectrogram analysis of infant crying using deep learning models offers a useful approach to distinguish between preterm and term infants and estimate GA variations, particularly in preterm infants. We identified that temporal fluctuations (i.e., contour and variance) in frequencies within the cry spectrogram are critical features that serve as acoustic markers reflecting fetal maturation and early neurodevelopmental processes. Additionally, the findings suggest that temporal structures in cry acoustics, particularly in the onset and offset regions of vocalizations, serve as key indicators of GA differences. Consequently, we argue for the importance of utilizing whole spectrograms that preserve temporal characteristics for a more nuanced prediction of individual differences in neurological development from infant cries.

By integrating deep-learning models that capture a broader range of acoustic features with longitudinal neurodevelopmental data in preterm infants, we would expect to further elucidate the acoustic patterns that contribute to the understanding of atypical early neurodevelopment. This approach may pave the way for the development of cry-based screening tools, improve risk detection, and enable early intervention strategies in clinical settings.

## Method

### Data availability

All associated simulation codes are accessible online through OSF (https://osf.io/sybv6/). For ethical reasons, we could not distribute the Mel-spectrograms of each crying infant. However, we have uploaded the embedding vectors of the model (NumPy file), which can be used to fully replicate our results.

### Participants

A total of 79 preterm neonates (GA < 37 weeks) and 52 term neonates (GA ≥ 37 weeks) comprised the study cohort at term-equivalent age. Recruitment occurred between 2011 and 2015 from the neonatal intensive care unit at Kyoto University Hospital, Japan, with infants around term-equivalent age (i.e., PMA ranging from 35 to < 42 weeks). These participants were partially incorporated into prior investigations conducted by our research team^5,7,14,17,43–45^. The inclusion criteria mandated the absence of severe neurological complications, including cerebral lesions (encompassing periventricular leukomalacia and Grade III or IV intraventricular hemorrhage) and chromosomal abnormalities. All participants came from Japanese families and were considered middle-class based on the census of their area of residence (Kyoto Prefecture). This study was conducted with the approval of the ethics committees of Kyoto University Graduate School and the Faculty of Medicine (No. E581) and the University of Tokyo (No. 23-521), in accordance with the standards specified in the 1964 Declaration of Helsinki. The demographic data of the participants at birth are presented in Table 2.

**Table 2:** Demographic variables in preterm and term infants.

	Preterm (*n* = 79)			Term (*n* = 52)		
	*M*	*SD*	*Range*	*M*	*SD*	*Range*
Gestational age (weeks)	31.7	3.6	23.1–31.9	39.2	1.2	37.0–41.4
Birth weight (g)	1462	303	618–1640	2960	376	2126–4110
Apgar score 5 min	7.8	2.1	1–10	9.3	0.5	8–10
IUGR	29/79 (37%)			2/52 (4%)		
Female	36/79 (46%)			24/52 (46%)		

*IUGR* intrauterine growth retardation.

### Cry recording

Written informed consent was obtained from the parents of all infants in the hospital before crying was recorded. The infants were around term-equivalent age (i.e., 35–45 weeks of PMA), and studied between 5 and 9 p.m. while in a supine position in an open crib. The preterm infants were recorded at 9–131 days after birth (chronological age [days]: *M* = 52.51, *SD* = 29.92; PMA [weeks]: *M* = 39.28, *SD* = 1.63), whereas term infants were recorded at 3–9 days after birth (chronological age [days]: *M* = 4.48, *SD* = 1.51; PMA[weeks]: *M* = 39.79, *SD* = 1.09). Significant group differences in chronological age was observed at cry recording but not in PMA. The difference was because preterm infants stayed longer, and some were recorded several times until they left the hospital (number of cry recordings: *M* = 1.67, *SD* = 0.92, *range* = 1–5). In contrast, term infants usually stayed within a week and were recorded only once. The authors’ previous studies analyzed only one cry within and closest to the term-equivalent age (i.e., 37–42 weeks of PMA), but the current study using deep learning expanded the period analyzed to include other cry recordings because a larger dataset may contribute to the classification of GA and group, and increase the generalizability of the findings.

Preterm infants were recorded in a growth care unit at Kyoto University Hospital, where they stayed until they left the hospital, whereas term infants were recorded in a quiet examination room. Environmental parameters, including the bassinet, auditory environment, and ambient temperature, were regulated for all participants. The auditory milieu of the rooms was deemed minimal and suitable for audio recording and analysis. Spontaneous cries of each infant, occurring no more than 30 min prior to feeding, were recorded for more than 60 s using a wave recorder at a 44.1 kHz sampling rate with 16-bit quantization. In cases where an infant was crying for longer than 60 s, a consecutive 60 s crying nearest to the feeding was selected. Throughout the recording, an approximate 15 cm span separated the microphone from the infant’s mouth (EDIROL R-09; Roland, Corp., Los Angeles, CA, United States).

### Cry analysis

Vocalization categorized as a cry utterance was delineated as an audible emission originating within a single exhalation and persisting for a time span of no less than 0.3 s. This definition was contrived to unequivocally exclude non-cry-related acoustic phenomena such as coughing^5^. Cry utterances were extracted manually using Praat (ver. 6.0.19)^46^. We manually segmented each infant’s 60 s cry series into single cry utterances based on amplitude-by-time waveforms. In total, 7306 cries were extracted, and those containing broad regions of environmental noise, such as the voices of other infants or medical staff, were excluded from the analysis to avoid artifacts. However, this study did not focus on *F*_*0*_ estimation; therefore, unlike previous studies focusing on *F*_*0*_ contours (e.g.^5,47^), we did not include whether *F*_*0*_ could be reliably determined with these signals by conventional methods in the inclusion criteria. Ultimately, 6523 cries (89.2% of all cry utterances) were used in the acoustic analyses (preterm group, *n* = 4698[*M* = 59.47, range = 10–226]; term group, *n* = 1825[*M* = 35.10, range = 4–67]). Because some of the preterm infants were measured several times, the mean number of cries analyzed per infant was higher in the preterm group than in the term group (*t* = 4.59, *p* < 0.001), but there was no significant group difference in the mean number of cries per measurement (*t* = 0.19, *p* = 0.847). The cry utterances were down-sampled to 22.05 kHz and low-pass filtered at 10 kHz when they were extracted in Praat.

The cry audio files were loaded into Jupyter Lab (Version 3.3.2, Version 3.9.16) using Librosa audio library functions. To process the feature extraction, Mel-spectrograms were extracted using Librosa’s speech extraction function (i.e., librosa.feature.Mel-spectrogram) with 1024 FFT points, 128 melbins, and a hop length of 256. Mel-spectrograms visually represent sound as perceived by humans by converting the Y-axis to a Mel scale, which demonstrates human sonic traits with a linear distribution below 1000 Hz and an increasing logarithmic scale above 1000 Hz. Based on the immaturity of early infant vocal tract control^2,21^, it is advantageous to employ Mel-spectrograms, which are highly sensitive to low-frequency sounds, for the classification of infant crying^27^.

### Classification of preterm versus term groups on CNN

#### CNN model

We utilized the base model VGG16 for feature extraction. The pretrained weights of VGG16 were loaded and set to be untrainable, leveraging transfer learning. Subsequently, a GlobalMaxPooling2D layer was incorporated to capture the most salient features (maximum values) from each channel. Readers may wonder why we inserted this GlobalMaxPooling layer, because it loses information on the inputs to some extent. Our pilot study revealed that including/excluding this layer did not change the classification performance of the model dramatically (although the accuracy was certainly higher without the GlobalMaxPooling layer—i.e., classification accuracy was 93.89%). By contrast, the computational resources and the time for training was drastically reduced by inserting this layer because the size of the resultant vector was reduced to approximately 2%. Without inserting the GlobalMaxPooling layer, most machines would not have sufficient computational resources to conduct training, which would have significantly affected the replicability of the current study. Therefore, we inserted a GlobalMaxPooling layer. This layer was succeeded by two dense layers, each consisting of 500 units, activated by a ReLU function and supplemented with a 25% dropout. A final dense layer with two units (softmax function) was added for binary classification (Fig. 1).

To optimize the computational efficiency and streamline training, we excluded the base portion of the model, where the weights were set as untrainableduring the training process. Specifically, we fed all the inputs (detailed in the subsequent section) into the base segment of the model. This resulted in the extraction of embeddings, which were the output vectors from the GlobalMaxPooling layer. The embeddings were stored (available online). In the training phase, only the top segment of the model, with its fully trainable weights, was constructed using the previously extracted embeddings as input.

#### Representations and data augmentation

The input tensor, representing 6523 Mel-spectrograms, had dimensions of 200 (height) × 300 (width) and spanned three channels (RGB). Values within this tensor were normalized to ensure that they ranged from zero to one. A thin white outer frame is observed within each Mel-spectrogram. Thus, the actual size of the Mel-spectrogram was approximately 180 (height) × 250 (width). The value of this white outer frame was set to zero in every Mel-spectrogram (essentially zero padding). To enhance the dataset, these 6523 three-dimensional tensors were augmented. Specifically, we augmented them ten-fold by superimposing three white patches, each of size 10 × 10, onto each Mel-spectrogram and setting the patch values to zero (i.e., 6523 original images and 58,807 images with three white patches).

#### Training and model evaluation (leave-one-out approach)

A leave-one-out cross-validation approach was employed. Specifically, all cry instances of a single infant (including augmented instances) were excluded from the training set. Only the original cry instances (excluding the augmented instances) from this excluded infant were used for the evaluation. This procedure was repeated 131 times, corresponding to the number of infants, to ensure that each infant was included at least once as part of the evaluation set. All results presented in the “Results” section are based on the performance of the model on this evaluation dataset.

The model was compiled using a stochastic gradient descent (SGD) optimizer with a learning rate of 0.0001, and a cross-entropy loss function was adopted. To address potential imbalances in the number of stimuli between the two categories, the loss was scaled by class weights. Specifically, the weight of each class was computed as the total number of stimuli divided by the product of the number of classes and the number of stimuli in that class. Consequently, this weighting scheme ensures that each class contributes equally to the loss, thus compensating for any discrepancies in the sample size between classes. The number of epochs was 200, and the batch size was 32.

The performance of the model for a given infant was then evaluated using the ratio of the number of correctly classified crying sounds to the total number of crying sounds from the test infant (i.e., the accurate classification rate; see Fig. 2). If this value was greater than 0.5, the model was considered to correctly classify the infant. We also obtained the precision, recall, F1 score, ROC curve, and precision–recall curves (Table 1 and Fig. 3).

#### Comparisons with the shuffle condition and the shuffle-and-slice condition

In the shuffle condition, the x-axis values (representing time) within each Mel-spectrogram were randomized, leading to a loss of frequency contour information over time across all the original 6523 Mel-spectrograms. For data augmentation, this shuffling procedure was repeated nine times, yielding an additional 58,807 images. Three white patches, each 10 × 10 in size, were superimposed onto the augmented images consistent with the original model. The training and evaluation approaches employing the leave-one-out method remained identical to those of the original model. For evaluation purposes, shuffled images (devoid of white patches) corresponding to the excluded infants were used.

For the shuffle and slice conditions, each of the original 6523 Mel-spectrograms underwent the aforementioned shuffling procedure. Subsequently, they were partitioned into one-tenth of the sections along the horizontal axis. This ensured that the total number of the resulting images matched that of the original model, amounting to 65,230 images (including augmented images). To maintain the input tensor dimensions (200 in height × 300 in width), a white rectangle (essentially 0-padding) was appended to the right side of each segmented image. The training and evaluation using the leave-one-out strategy paralleled the approach of the original model. During the evaluations, all shuffled and sliced images associated with the omitted infants were employed. Under these conditions, the rationale for selecting a specific slice size was not robust. Hence, an alternative model was tested in which each Mel-spectrogram was divided into one-twentieth sections. The classification accuracy remained consistent with that of one-tenth of the segmentation approach. Consequently, we present the one-tenth of the segmentation results in the main text (Figs. 2 and 3).

### Grad-CAM: highlighting key features within Mel-spectrograms

We used a Grad-CAM technique^34^. All analytical codes are available online at OSF (https://osf.io/sybv6/). Grad-CAM computes the gradient of the predicted class score with respect to the feature maps of a given convolutional layer, and essentially identifies the importance of each feature map for the predicted class. After 200 training epochs in the original model, all Mel-spectrogram images of the excluded infants were used for evaluation. Subsequently, the activity and loss of the last convolution layer were probed only in correctly classified trials. The gradients of the predicted class score with respect to the feature maps of the target layer provide a measure of the importance of each feature map for the predicted class. We obtained a channel-wise means of these gradients, resulting in a set of weights. Each feature map of the target layer is weighted using its corresponding gradient-derived weight. The weighted feature maps were then combined by taking their weighted sum to produce a coarse heat map. This heat map highlights the important regions in the image for the predicted class. The heatmap values were normalized between 0 and 255 and subsequently converted to a colormap representation for better visualization. The color map was then superimposed on the original Mel-spectrogram to highlight the crucial areas influencing the prediction of the model. The representative outcomes (left) and the average of all outcomes (right) are shown in Fig. 4.

### Prediction of GA based on LSTM network

#### LSTM network model

The architecture of the model is similar to that of the aforementioned CNN model. Comprising the VGG16 architecture with untrainable weights, followed by a GlobalMaxPooling layer (see Fig. 5). For each infant, every Mel-spectrogram (200 × 300 × 3 in RGB) was sequentially fed into the base part of the model in the chronological order of crying episodes. The resulting output vectors or embeddings were aggregated to construct a two-dimensional matrix for each infant, with dimensions representing 226 (maximum number of cry instances) × 512 (embedding vector units). In cases where the number of cry instances for a specific infant did not reach the dataset’s maximum count of 226, we padded the sequence with a value of − 1 to maintain consistent matrix dimensions across all infants. This padded sequence was masked during the training.

During the training process, the two-dimensional matrix was inputted into an LSTM layer consisting of 30 units designed to process and aggregate 226 sequential embeddings (maximally). This was followed by a dropout layer that inactivated 20% of the units to prevent overfitting. Subsequently, a dense layer of 30 units and rectified linear unit activation function were added. The final output of the model produced a single estimate of the GA value. The target GA value was linearly normalized to fit within the range of 0–1. Consequently, the activation function employed for the final output layer was a sigmoid function.

The leave-one-out methodology was adopted for training and evaluation. In this approach, the two-dimensional matrix (comprising 226 time sequences by 512 embeddings) of 130 infants served as the training set, whereas the matrix from a single excluded infant was utilized for evaluation. This procedure was performed 131 times to ensure that each infant was individually selected as an evaluation sample. During each iteration, the training comprised 150 epochs with a batch size of 16. The loss function employed was mean squared errors, and the optimization was carried out using the ‘Adam’ algorithm. Data augmentation is not implemented in the LSTM network simulation.

## Data Availability

Data availability statement The source codes that support the findings of this study are openly available in Open Science Framework at https://osf.io/sybv6/.
